# Contraceptive use and mental health among women of reproductive age: insights from the Mozambique Demographic and Health Survey

**DOI:** 10.1192/bji.2025.10045

**Published:** 2026-05

**Authors:** Emmanuel Anongeba Anaba, Melissa E. Wright, Stanley Kofi Alor, Madison Lindeman, Yula Salifu, Samuel Adjorlolo, Adom Manu

**Affiliations:** 1 Department of Population, Family and Reproductive Health, School of Public Health, University of Ghana, Legon, Ghana; 2 Cardiff University Brain Research Imaging Centre (CUBRIC), School of Physics and Astronomy, Cardiff University, Cardiff, UK; 3 Ghana Armed Forces College of Nursing and Midwifery 37 Military Hospital, Accra, Ghana. Email: alorstanley@gmail.com; 4 School of Geographical Sciences, University of Bristol, Bristol, UK; 5 Department of Population, Family and Reproductive Health, School of Public Health, University of Ghana, Legon, Ghana; 6 Department of Mental Health, School of Nursing and Midwifery, University of Ghana, Legon, Ghana; 7 Department of Population, Family and Reproductive Health, School of Public Health, University of Ghana, Legon, Ghana

**Keywords:** Contraceptive use, depression, anxiety, demographic and health survey, Mozambique

## Abstract

**Background:**

Contraceptive use among women of reproductive age has increased significantly worldwide. However, little is known about contraceptive use and mental health among women in sub-Saharan African countries.

**Aims:**

This study sought to investigate contraceptive use and self-reported measures of depression and anxiety symptoms among reproductive-age women in Mozambique using the most recent national data.

**Method:**

The study used secondary data from the 2022–2023 Demographic and Health Survey of Mozambique. A total of 6910 (weighted) sexually active women aged 15–49 years were included in this analysis. Anxiety and depression scores were measured using self-report questionnaires. Linear regression analysis was conducted to assess the associations between depression and anxiety and contraceptive use.

**Results:**

The prevalence of contraceptive use was 36.41%. About half of the hormonal contraceptive users (49.38%) were using injectables and 25.99% were using implants. In total, 9.14 and 2.83% of the women had moderate or high scores of self-reported anxiety respectively. Additionally, 5.24 and 5.42% reported moderate or high scores of depressive symptoms respectively. The use of hormonal contraceptives decreased anxiety symptoms by 0.47 units (*β* = −0.47; 95% CI −0.75 to −0.18; *P* < 0.001) compared with no contraceptive use. The use of hormonal contraceptives decreased depressive symptoms by 0.75 units (*β* = −0.75; 95% CI −1.11 to −0.39; *P* < 0.001) compared with no contraceptive use.

**Conclusions:**

This study has demonstrated a naturalistic association between contraceptive use, particularly hormonal contraceptives, and decreased anxiety and depression among Mozambican women of reproductive age. This likely reflects a complex, bi-directional relationship, the nature and mechanism of which should be investigated in further experimental research.

The use of contraceptives by women of reproductive age has greatly increased worldwide.^^1^^ Contraceptive use allows women to take control of their reproduction by reducing the risk of unwanted pregnancies and the associated psychosocial, financial, cultural and health burdens.^2^ A range of contraceptives are available: hormonal and non-hormonal, long- and short-term, and one-time barriers.^3^ Anxiety and depression symptoms can be debilitating and show a higher prevalence in women than in men.^4,5^ There is evidence that contraceptive use has a protective effect against depression and anxiety, particularly in women who have a history of mental disorders, although significant heterogeneity across results and methodologies is apparent.^6^ Additionally, the impact of contraceptive use on mental health appears to differ by country,^7–9^ possibly owing to varying sociocultural contexts and health factors (e.g. nutrition, vascular health, endocrine health).

Little is known about contraceptive use and mental health in sub-Saharan African countries. The rate of modern contraceptive use by Mozambican women of reproductive age is relatively low, but has been slowly increasing over recent years, from 13% in 2003 to 20% in 2015^10,11^ and 26% in 2022,^12^ reflecting the global emphasis on family planning as a vital component of family health, well-being and socioeconomic development.^13^ However, the relationship between contraceptives and mental health (whether beneficial or detrimental) in Mozambican women has not been investigated. To understand the potential impact of contraceptive use on African women’s mental health, it is vital to first examine whether there is evidence for a relationship. Such a relationship can then be further interrogated in follow-up studies to tease apart directionality and mechanisms. To the best of our knowledge, this is the first study to investigate, using nationally representative data, whether hormonal and non-hormonal contraceptive use is associated with differences in depressive and/or anxiety symptom levels compared with not using contraceptives among reproductive-age women in Mozambique.

## Method

### Study setting and design

Mozambique is a lower-middle-income country located in Southeast Africa. It has an estimated population of over 33 million, of which about 17 million are female.^14^ The 2022–2023 Mozambique Demographic and Health Survey (MDHS) was a household-based survey conducted across all the administrative regions of the country.^15^ The MDHS collected data on demographic and health indicators, including family planning, depression and anxiety.

### Target population and sampling

The MDHS focused on women of reproductive age (15–49 years), including rural and urban residents. The survey used a two-stage cluster sampling technique. In the first stage, clusters were selected from a sampling frame. In the second stage, all households within the selected clusters were listed and some were selected using a systematic sampling technique proportional to size. All women who were permanent residents of the selected households were eligible for the survey. In the 2022–2023 MDHS, a total of 13 183 women aged 15–49 years were interviewed. The study described here focused on women aged 15–49 years who were at risk of getting pregnant: thus, women who were sexually active during the 30 days before the date surveyed (i.e. in the previous 30 days). Therefore, women who never had sex (*n* = 1222), had been sexually inactive in the previous 30 days (*n* = 4616) and those currently pregnant (*n* = 714) were excluded. A total of 6631 (unweighted) or 6910 (weighted) sexually active women aged 15–49 years were included in this study.

### Outcome variables

The dependent variables for this study were depression and anxiety, measured using the self-reported Patient Health Questionnaire (PHQ-9) and Generalized Anxiety Disorder scale (GAD-7) respectively. The GAD-7 has seven items with scores ranging from 0 to 21, and the PHQ-9 has nine items with scores ranging from 0 to 27. Each symptom in both scales is assigned a score (0 = never; 1 = rarely; 2 = often; or 3 = always) depending on how frequently the respondent reported experiencing the symptom in the previous 2 weeks. Higher scores are associated with more severe symptoms of depression and anxiety.^16^

### Independent variable

Contraceptive use was the independent variable. The women were asked whether they had tried in any way to delay or avoid getting pregnant. A follow-up question was asked about their current contraceptive method (no method = 0; hormonal method = 1; non-hormonal method = 2). We adjusted for covariates, including sociodemographic characteristics (age, education, wealth index quintile, religion, marital status, parity and place of residence), exposure to family planning information on television and radio, and knowledge about contraception. We also adjusted for suicidal ideation and suicide attempts in the previous 12 months and history of pregnancy termination. Details about these items are provided elsewhere.^15^

### Statistical analysis

The MDHS data were downloaded, recoded and analysed using STATA/SE version 17 for Windows. Sampling weights, clustering and stratification (complex survey design) were taken into consideration during the analysis. We adjusted for the complex survey design using the ‘svy’ command in STATA. Descriptive statistics were computed for participant characteristics, contraceptive use, depression and anxiety. Both simple and multiple linear regression analyses were conducted to assess the association between contraceptive use and depression and anxiety in the sample. Variables that were statistically significant in the simple linear regression analysis were adjusted in the multiple linear regression model. Statistical significance was considered at *P* < 0.05 and adjusted *β*-coefficients were reported at a 95% confidence interval.

### Ethical standards

The authors assert that all procedures contributing to this work comply with the ethical standards of the relevant national and institutional committees on human experimentation and with the Helsinki Declaration of 1975, as revised in 2013. The 2022–2023 MDHS protocol was approved by the National Bioethics Committee for Health in Mozambique (CNBS) and the ICF Institutional Review Board. Written informed consent was obtained from all MDHS participants, guardians and parents. Ethical approval was not required for this study since it is a further analysis of the de-identified data from the MDHS. We downloaded the data-set after receiving email approval from the Demographic and Health Surveys (DHS) Program.

## Results

### Participant characteristics

Participant characteristics are shown in Table 1. The mean age of the participants was 30.15 years (s.d. = 0.17 years) and about 30% had no formal education. About 26% of participants were in the ‘richest’ wealth index quintile, seven in ten professed Christianity (72.16%) and more than half were married or in a union with a man (80.55%). Over 50% of participants resided in rural areas (59.90%) and 67.88% were not working at the time of the survey. More than 90% of participants knew about family planning methods, with 26.89 and 31.32% exposed to family planning information on television and radio respectively. The prevalence of suicidal ideation and suicide attempts in the previous 12 months was about 4 and 2% respectively. In total, 11.63% of participants had ever terminated a pregnancy and the average number of children ever born was 3 (s.d. = 0.05). Regarding contraceptive use, less than half of participants were using contraception (36.41%), with 30.2% using hormonal contraception. Among participants who were using hormonal contraceptives, about half (49.38%) used injectables, 25.99% used implants, 19.81% used pills and 4.82% used other hormonal methods.

**Table 1:** Participant characteristics

Characteristic	*n*	%
Contraceptive use		
No method	4394	63.59
Hormonal	2087	30.20
Non-hormonal	429	6.21
Wealth index		
Poorest	1182	17.11
Poorer	1340	19.39
Middle	1192	17.26
Richer	1406	20.34
Richest	1790	25.90
Religion		
Christianity	4986	72.16
Islam	1408	20.37
No religion	516	7.47
Educational status		
None	2056	29.76
Primary	2793	40.42
Secondary	1839	26.62
Higher	222	3.20
Place of residence		
Rural	4139	59.9
Urban	2771	40.1
Marital status		
Never in a union	803	11.62
In a union/married	5566	80.55
Divorced/separated/widowed	541	7.83
Currently working		
No	4690	67.88
Yes	2220	32.12
Exposure to family planning on television		
No	5052	73.11
Yes	1858	26.89
Exposure to family planning on the radio		
No	4746	68.68
Yes	2164	31.32
Knowledge of family planning methods		
Knows no method	263	3.80
Knows a method	6647	96.20
Terminated a pregnancy		
No	6106	88.37
Yes	804	11.63
Suicidal ideation in previous 12 months		
No	6554	94.85
Yes	2745	3.97
Refused to answer	81	1.18
Suicide attempt in previous 12 months		
No	6759	97.82
Yes	123	1.77
Refused to answer	28	0.41
Age, years: mean (s.d.)	30.15 (0.17)	
Number of children: mean (s.d.)	3 (0.05)	

### Prevalence of self-reported depression and anxiety symptoms

The majority of participants reported no/minimal depression symptoms on the PHQ-9, and 18.3, 5.24 and 5.42% reported low, moderate and high scores respectively. On the GAD-7, 65.33% of participants reported minimal anxiety symptoms; 22.7, 9.14 and 2.83% had low, moderate and high reported scores respectively.

### Factors associated with self-reported depression and anxiety symptoms

The results of the simple linear regression analysis for depression and anxiety symptoms are given in the Supplementary material, available online at https://doi.org/10.1192/bji.2025.10045. The analysis showed that depression scores had a statistically significant association with contraceptive use, wealth index quintile, religion, educational status, currently working, exposure to family planning information on television, pregnancy termination, suicidal ideation and suicide attempts. For instance, hormonal contraceptive use (*β* = −1.19; 95% CI −1.58 to −0.79) was associated with a 1.19 unit decrease in depression score compared with no contraceptive use. Depression decreased by 1.64 units among women in the ‘richest’ wealth index quintile (*β* = −1.64; 95% CI −2.50 to −0.78) compared with those in the ‘poorest’ quintile. Women with higher educational status (*β* = −1.43; 95% CI −2.16 to −0.69) had a 1.43 unit decrease in depression compared with those with no formal education. Additionally, women who had had suicidal ideation (*β* = 2.19; 95% CI 1.27– 3.12) in the previous 12 months had a 2.19 unit increase in depression compared with those who had no suicidal ideation. Similarly, a suicide attempt (*β* = 2.64; 95% CI 1.30–3.98) in the previous 12 months was associated with a 2.64 unit increase in depression compared with no suicide attempt.

The simple linear regression analysis also showed a statistically significant relationship between anxiety scores and contraceptive use, wealth index quintile, religion, educational status, work status, suicidal ideation and suicide attempt. For example, hormonal contraceptive use was associated with a 0.78 unit decrease in anxiety score compared with no contraceptive use (*β* = −0.78; 95% CI −1.10 to −0.46). Moreover, anxiety decreased by 0.98 units among women in the ‘richest’ wealth index quintile (*β*= −0.99; 95% CI= −1.62 to −0.34) compared with those in the ‘poorest’ quintile. Women with higher educational status (*β* = −0.69; 95% CI= −1.31 to −0.06) had a 0.69 unit decrease in anxiety compared with those without formal education. Additionally, women who had had suicidal ideation (*β* = 1.14; 95% CI 0.33–1.94) in the previous 12 months had a 1.14 unit increase in anxiety compared with those who had had no suicidal ideation. Similarly, a suicide attempt (*β* = 1.68; 95% CI 0.61– 2.76) in the previous 12 months was associated with a 1.69 unit increase in anxiety compared with no suicide attempt.

### Multiple linear regression of factors associated with anxiety

In the adjusted linear regression model, hormonal contraceptive use and currently working were associated with decreased anxiety levels (Table 2). Religion, suicidal ideation and attempted suicide were associated with increased anxiety levels. The use of hormonal contraceptives decreased anxiety symptoms by 0.47 units (*β* = −0.47; 95% CI −0.75 to −0.18) compared with no contraceptive use. Also, participants who were working had a decreased anxiety score by 0.47 units (*β* = −0.47; 95% CI −0.75 to −0.18). Moreover, not professing any religion and refusing to answer the survey questions about suicidal ideation and suicide attempts increased the anxiety level by 1.48 units (*β* = 1.47; 95% CI 0.80– 2.15), 7.51 units (*β* = 7.51; 95% CI 6.11– 8.91) and 1.46 units (*β* = 1.45; 95% CI 0.28– 2.63) respectively.

**Table 2:** Multiple linear regression of factors associated with anxiety among women of reproductive age

Variable	*β* coefficient	95% CI	*P*
Contraceptive use (reference: no method)			
Hormonal	−0.47	−0.75 to −0.18	0.001
Non-hormonal	−0.17	−0.79 to 0.44	0.571
Wealth index (reference: poorest)			
Poorer	−0.56	−1.23 to 0.10	0.096
Middle	−0.58	−1.26 to 0.09	0.091
Richer	−0.41	−1.13 to 0.31	0.263
Richest	−0.56	−1.21 to 0.08	0.090
Religion (reference: Christianity)			
Islam	0.45	−0.017 to 0.92	0.059
No religion	1.47	0.80 to 2.15	<0.001
Educational status (reference: none)			
Primary	0.19	−0.24 to 0.63	0.378
Secondary	0.32	−0.13 to 0.79	0.169
Higher	−0.08	−0.86 to 0.69	0.832
Currently working (reference: no)			
Yes	−0.47	−0.75 to −0.18	0.001
Suicidal ideation in previous 12 months (reference: no)			
Yes	0.66	−0.25 to 1.57	0.156
Refused to answer	7.51	6.11 to 8.91	<0.001
Suicide attempt in previous 12 months (reference: no)			
Yes	1.45	0.28 to 2.63	0.015
Refused to answer	−0.37	−4.27 to 3.52	0.850

### Multiple linear regression of factors associated with depression

In the multiple linear regression analysis, depression was significantly associated with contraceptive use, wealth index quintile, currently working, religion, suicidal ideation and suicide attempts (Table 3). The use of hormonal contraceptives decreased depressive symptoms by 0.75 units (*β* = −0.75; 95% CI −1.11 to −0.39) compared with no contraceptive use. Also, those who were currently working had a decreased depression score by 0.37 units (*β* = −0.37; 95% CI −0.68 to −0.06). Furthermore, those in the ‘middle’ and ‘highest’ wealth quintiles had a decreased depression score by 0.97 units (*β* = −0.97; 95% CI −1.84 to −0.09) and −1.00 units (*β* = −1.00; 95% CI −1.91 to −0.09) respectively. Moreover, professing Islam, having suicidal ideation, refusing to answer the survey questions about suicidal ideation and have made suicide attempts increased the depression score by 1.05 units (*β* = 1.05; 95% CI 0.38–1.71), 1.60 units (*β* = 1.60; 95% CI 0.53–2.67), 8.87 units (*β* = 8.87; 95% CI 6.96– 10.79) and 1.79 units (*β* = 1.79; 95% CI 0.28–3.29) respectively.

**Table 3:** Multiple linear regression of factors associated with depression among participants

Depression	*β* coefficient	95% CI	*P*
Contraceptive (reference: no method)			
Hormonal	−0.75	−1.11 to −0.39	<0.001
Non-hormonal	−0.51	−1.10 to 0.08	0.090
Wealth index (reference: poorest)			
Poorer	−0.66	−1.55 to 0.21	0.139
Middle	−0.97	−1.84 to −0.09	0.029
Richer	−0.48	−1.44 to 0.47	0.322
Richest	−1.00	−1.91 to −0.08	0.032
Religion (reference: Christianity)			
Islam	1.04	0.38,1.71	0.002
No religion	−0.34	−0.96 to 0.27	0.273
Educational status (reference: none)			
Primary	0.11	−0.38 to 0.61	0.653
Secondary	0.00	−0.46 to 0.46	0.997
Higher	−0.60	−1.50 to 0.30	0.191
Currently working (reference: no)			
Yes	−0.37	−0.67 to −0.06	0.019
Exposure to family planning messages on television (reference: no)			
Yes	0.24	−0.21 to 0.71	0.297
Terminated pregnancy (reference: no)			
Yes	−0.28	−0.68 to 0.11	0.158
Suicidal ideation in previous 12 months (reference: no)			
Yes	1.60	0.53 to 2.67	0.003
Refused to answer	8.87	6.95 to 10.78	<0.001
Suicide attempt in previous 12 months (reference: no)			
Yes	1.78	0.27 to 3.29	0.020
Refused to answer	0.02	−5.50 to 5.56	0.992

## Discussion

This study aimed to investigate the relationship between contraceptive use and metrics of mental health in a representative naturalistic sample of reproductive-age women in Mozambique. It was found that hormonal contraceptive use was associated with a decrease in self-reported depression and anxiety scores compared with no contraceptive use.

This is the first study to examine whether contraceptive use is associated with mental health in a sub-Saharan African population. Previous studies have investigated this in other cultures and reported inconsistent results. For example, in a North American sample, Gawronska et al reported that hormonal contraceptive users had lower depressive symptoms than non-users.^7^ However, in a Saudi Arabian sample, hormonal contraceptive methods were associated with an increase in the risk of moderate-to-severe depression compared with non-hormonal methods.^17^ A recent systematic review of randomised controlled trials and cohort studies from a range of countries (mostly the Americas and Eurasia) found evidence for a protective effect of hormonal contraceptives on anxiety and, for those with a history of mental illness, on depression.^6^ These cross-cultural differences may reflect the complex bi-directional relationship between contraceptive use and mental health. This relationship is likely to differ between specific populations owing to factors such as cultural perceptions regarding contraception and fertility (e.g. fears that contraception may lead to infertility), social support (from immediate family or the wider community) and general sexual and reproductive health that may be influenced by factors relating to the social determinants of health. This is evident in other areas relating to sexual and reproductive health: for example, a range of factors, including sociocultural context, have been shown to have an impact on menstrual experiences.^18^ It is therefore vital to investigate this topic in specific communities, such as Mozambique.

This study presents the first finding of a relationship between mental health and contraceptive use in a naturalistic sample of Mozambique women. Further work should now fully investigate the mechanisms behind this relationship, especially the direction and causality. It is likely that it is a complex, bi-directional relationship. For example, those with lower self-reported anxiety and depression scores may be more able to access contraceptives. Equally, hormonal contraceptives themselves may lead to improved mental health, either owing to the freedom, control and empowerment offered by their use, or by direct neuroendocrine action.

The socioeconomic benefits of hormonal contraception have been widely cited throughout the literature. They include preventing unwanted pregnancy^19–21^ and improving menstrual disorders such as heavy menstrual bleeding, pelvic pain and endometriosis.^20–23^ This reduction in the health and social issues surrounding unwanted pregnancy and menstrual disorders may be a potential pathway by which contraceptive use leads to better mental health and well-being outcomes, especially as such menstrual disorders can have a negative impact on the quality of life.^24,25^ This may be exacerbated by lack of easily accessible sexual and reproductive health education and menstrual products in certain areas of Africa,^26^ so women in these regions may particularly benefit from contraceptive use. Further research should tease apart these contributing factors.

Multiple covariates were added to these models to adjust for their influence on anxiety and depression scores outside of contraceptives. From our analysis, a number of these appear particularly influential on self-reported mental health metrics. It was evident that both anxiety and depression were significantly associated with religion, work status, suicidal ideation and suicide attempts. Women with no religion had increased anxiety scores, while those who professed Islam had increased depression scores. This points to a nuanced interplay between religious affiliation and mental health outcomes among women. These findings align with those of Antable and colleagues,^27^ who also found that Mozambican women who professed Islam had higher odds of depression, while those who had no religious affiliation had higher odds of anxiety. The duality may be explained by several factors, including lack of social support systems, gender-specific norms and internalised religious coping styles. For instance, women who have no religious affiliation may lack structured social support and coping frameworks, leading to poor mental health outcomes.^28^ On the other hand, Muslim women may face gender-specific restrictions (menstruation-related taboos and stigma), and navigating these may intensify psychological distress.^29^ Additionally, negative religious coping, religious strain and religious guilt may worsen mental health vulnerabilities.^30^

Employment status also had a significant association with self-reported mental health scores; women who were currently working experienced a reduction in both anxiety and depression. Employment can provide financial security and a sense of purpose, and foster connections between individuals. Our findings are in line with those of others, such as Lund et al, who conducted two systematic reviews in low- and middle-income countries.^32^ They called for increased interventions to alleviate poverty, noting that poverty and poor mental health interact in a negative cycle. This is also a potential pathway for the relationship between contraceptives and mental health in Africa: women with more control over their fertility and reproductive health may have more stable employment, which in turn improves mental health. Similarly, our results highlight that women in the ‘middle’ and ‘richest’ wealth index quintiles were less likely to experience depression than those with other socioeconomic statuses. As previously discussed, income and financial security are associated with reduced depression.^33^ Other external factors may be influencing the prevalence of depression among women in the ‘richer’ quintile, such as access to mental health services.

### Implications

The findings of this study have several implications for family planning, policy, healthcare programes and research. The suboptimal use of contraception among sexually active women in Mozambique is a major concern and requires urgent attention from stakeholders. Health service providers must intensify efforts to increase contraceptive uptake among women at risk of getting pregnant. Following a more thorough investigation, interventions such as family planning education could highlight the mental health benefits associated with hormonal contraceptives. Indeed, research on this topic enables women to be fully informed when choosing contraceptives. Additionally, anxiety and depression interventions among sexually active women in Mozambique should prioritise women of low socioeconomic status and those with a history of suicidal behaviours. The complex bi-directional relationship between hormonal contraception and mental health requires further research, especially among women in sub-Saharan African countries.

### Strengths and limitations

A major strength of this study is the use of recent nationally representative data in a rarely studied population; hence, the findings can be generalised to sexually active women in Mozambique and may be indicative of populations in other sub-Saharan African countries. The data analysed in this study were collected using rigorous methods, including robust sampling techniques and standard questionnaires, which improves the validity and reliability of the findings. However, interpretation is limited by the cross-sectional nature of the study. Cross-sectional studies cannot confirm the direction or causality of relationships. Therefore, future research is needed to untangle this relationship in this population. As mentioned above, it is likely that this is a bi-directional relationship, but this requires direct investigation.

Furthermore, associations were not made with clinically rated symptoms or diagnoses, but with self-reported questionnaires probing levels of depression and anxiety. This is useful for assessing indicators of mental health in non-clinical populations, but it must be kept in mind that it may not translate to clinical manifestations or diagnoses. Additionally, more detailed information, such as exact hormones included, duration of contraceptive use, previous hormonal or anti-depressant medication, and detailed mental health history, was not provided but could lead to further insights into this relationship.

## Supporting information

Anaba et al. supplementary material Anaba et al. supplementary material

## Data Availability

The data used in this study may be requested from the data owners, the Demographic and Health Surveys (DHS) Program (https://dhsprogram.com/data/available-datasets.cfm). The authors confirm they did not have any special access or privileges to the data that other researchers would not have.
