# Supplementary material for: Contraceptive use and mental health among women of reproductive age: insights from the Mozambique Demographic and Health Survey
Source: BJPsych Int. 2025 Jul 11;23(2):61–7. doi: 10.1192/bji.2025.10045 (PMC13732270; doi:10.1192/bji.2025.10045)
Supplement: Supplementary file 1 [file S2056474025100457sup001.pdf]

Supplemental material

Table 2: Simple linear regression of factors associated with depression among women of reproductive age

Depression	Coefficient	95% CI	p-value
**Contraceptive (reference: no method)**			
Hormonal	-1.19	-1.58, -0.79	< 0.001
Non-hormonal	-1.10	-1.71, 10.46	< 0.001
**Age of respondent**	0.01	-0.00, 0.02	0.229
**Wealth index(reference: poorest)**			
Poorer	-0.75	-1.66, 0.15	0.105
Middle	-1.14	-2.01, -0.28	0.010
Richer	-0.65	-1.60, 0.29	0.177
Richest	-1.64	-2.50, -0.78	< 0.001
**Religion (reference: Christianity)**			
Islam	1.49	0.78, 2.20	< 0.001
No religion	-0.08	-0.81. 0.64	0.818
**Education (reference: none)**			
Primary	-0.11	-0.58, 0.34	0.627
Secondary	-0.66	-1.12, -0.21	0.004
Higher	-1.43	-2.16, -0.69	< 0.001
**Place of residence (reference: rural)**			
urban	-0.25	-0.78, 0.28	0.357
**Marital status (reference: never in union)**			
In union	0.46	-0.00, 0.92	0.050
Divorced/widowed/separated	0.74	-0.00, 1.49	0.052
**Currently working (reference: no)**			
Yes	-0.94	-1.31, -0.57	< 0.001
Number of children	0.02	-0.03, 0.08	0.438
**Exposure to FP messages on television (reference: no)**			
Yes	-0.71	-1.10, -0.33	< 0.001
**Exposure to FP messages on the radio (reference: no)**			
Yes	-0.15	-0.59, 0.28	0.480
**Knowledge of FP (reference: knows no method)**			
Knows a method	-0.14	-1.03, 0.75	0.758
**Terminated a pregnancy (reference: no)**			
Yes	-0.61	-1.05, -0.18	0.006
**Suicidal ideation in the last 12 months (reference: no)**			
Yes	2.19	1.27, 3.12	< 0.001
Refuse to answer	9.44	7.16, 11.73	< 0.001
**Suicidal attempt in the last 12 months (reference: no)**			
Yes	2.64	1.30, 3.98	< 0.001
Refuse to answer	6.40	1.56, 11.24	0.010

Table 3: Simple linear regression of factors associated with anxiety among women of reproductive age

Anxiety	Coefficient	95% CI	p-value
**Contraceptive (reference: no method)**			
Hormonal	-0.78	-1.10, -0.46	< 0.001
Non-hormonal	-0.57	-1.18, 0.03	0.064
**Age of respondent**	0.01	-0.00, 0.03	0.091
**Wealth index (reference: poorest)**			
Poorer	-0.62	-1.32, 0.07	0.079
Middle	-0.69	-1.39, 0.00	0.050
Richer	-0.46	-1.20, 0.28	0.225
Richest	-0.98	-1.62, -0.34	0.003
**Religion (reference: Christianity)**			
Islam	1.33	0.73, 1.93	< 0.001
No religion	-0.28	-0.80, 0.23	0.289
**Education (reference: none)**			
Primary	0.03	-0.39, 0.45	0.888
Secondary	-0.15	-0.60, 0.29	0.495
Higher	-0.69	-1.31, -0.06	0.031
**Place of residence (reference: rural)**			
urban	-0.02	-0.48, 0.43	0.907
**Marital status (reference: never in union)**			
In union	0.39	-0.00, 0.80	0.054
Divorced/widowed/separated	0.53	-0.08, 1.14	0.090
**Currently working (reference: no)**			
Yes	-0.84	-1.16, -0.51	<0.001
**Number of children**	-0.01	-0.07, 0.03	0.528
**Exposure to FP messages on television (reference: no)**			
Yes	-0.16	-0.50, 0.17	0.350
**Exposure to FP messages on the radio (reference: no)**			
Yes	0.30	-0.04, 0.65	0.091
**Knowledge of FP (reference: knows no method)**			
Knows a method	0.63	-0.15, 1.42	0.116
**Terminated a pregnancy (reference: no)**			
Yes	-0.29	-0.71, 0.12	0.168
**Suicidal ideation in the last 12 months (reference: no)**			
Yes	1.14	0.33, 1.94	0.005
Refuse to answer	7.91	6.12, 9.70	<0.001
**Suicidal attempt in last 12 months (reference: no)**			
Yes	1.68	0.61, 2.76	0.002
Refuse to answer	5.06	1.55, 8.57	0.005
